# Prothrombotic profile in patients with vasospastic or non vasospastic angina and non significant coronary stenosis

**DOI:** 10.1186/1477-9560-9-10

**Published:** 2011-05-27

**Authors:** Jaume Figueras, Jasone Monasterio, Enric Domingo, Beatriz Meneses, Elsa Nieto, Josefa Cortadellas, David Garcia-Dorado

**Affiliations:** 1 Unitat Coronària, Àrea del Cor, Laboratori d'Hemostàsia*, Hospital General Vall d'Hebron, Universitat Autònoma de Barcelona, Barcelona. Spain

## Abstract

**Background:**

Patients with vasospastic (VA) or non vasospastic angina (NVA) without significant coronary stenosis have a reduced risk of infarction but is unclear whether or not this may be attributable to a lack of prothrombotic profile - similar to that present in patients with stable coronary artery disease (CAD).

**Methods:**

Plasma levels of von Willebrand factor, total and free tissue factor pathway inhibitor, plasminogen activator inhibitor-1, and fibrinogen were analyzed in 15 patients with stable VA and 23 with NVA, all with vasoconstrictive response to acetylcholine although with different severity. Results were compared with those of 20 age-matched controls and 10 patients with CAD.

**Results:**

Plasma levels of von Willebrand factor in patients with VA or NVA were higher than in controls (207 ± 62 and 203 ± 69% vs 121 ± 38%, p < 0.001) and tended to be lower than in CAD patients (264 ± 65, p = 0.145). They also presented higher total tissue factor pathway inhibitor (123 ± 18 and 111 ± 25 vs 88 ± 14, ng/ml p < 0.001) and plasminogen activator inhibitor-1 levels than controls (51 ± 30 and 52 ± 31% vs 19 ± 9 ng/ml, p < 0.001) and similar to CAD patients (134 ± 23 and 62 ± 31, respectively, ns). Moreover, free tissue factor pathway inhibitor plasma levels were lower than controls (18 ± 5 and 17 ± 5 vs 23 ± 8 ng/ml, p = 0.002) and similar to CAD patients (14 ± 5, ns). Despite this prothrombotic condition none of VA or NVA patients presented a myocardial infarction during a 9 year follow-up, an observation also reported in larger series.

**Conclusions:**

During a stable phase of their disease, patients with VA or NVA present a prothrombotic profile that might eventually contribute to occurrence of myocardial infarction. The rarity of these events, however, may suggests that ill defined factors would protect these patients from coronary plaque rupture/fissure.

## Background

Endothelial dysfunction has been documented in patients with angina and non significant coronary stenosis, either vasospastic (VA)[1] or non vasospastic (NVA) - including those with syndrome X [2,3]. Overall, endothelial dysfunction appears to be a relevant event for it is often the first step of atherosclerosis [4,5] and may facilitate coronary thrombosis [6,7]. In fact, patients with atherosclerosis may present a protrombotic state characterized by an imbalance in the thrombotic-fibrinolytic equilibrium with abnormal plasma levels of von Willebrand factor (vWF)[8], tissue factor or tissue factor pathway inhibitor (TFPI)[9-11], plasminogen activator inhibitor-1 (PAI-1)[12-15] and fibrinogen [14,16]. In keeping with this, patients with unstable coronary artery disease and significant coronary stenosis often exhibit an abnormal coagulation profile which is associated with increased risk of coronary thrombotic events [14,17]. Nevertheless, VA and NVA patients and for unknown reasons only rarely develop a myocardial infarction [18-25]. Thus, the aim of this study was to investigate whether this reduced incidence of coronary thrombosis could in part be related to a lack of a pro-thrombotic profile despite a proven coronary endothelial dysfunction. Thus, we investigated plasma levels of relevant prothrombotic markers such as vWF, TFPI, PAI-1 and fibrinogen in stable patients with VA or NV. We compare these levels with those of patients with stable coronary artery disease (CAD) and with normal subjects.

## Methods

### Patients selection

Thirty eight consecutive patients with typical angina at rest responsive to sublingual nitroglycerin bsut without significant coronary stenosis (<50%) were selected from our chest pain out patient clinic. There were 15 with documented transient ST elevation and spontaneous and/or an ergonovine-induced coronary vasospasm categorized as VA patients, and 23 without ECG changes during pain and a negative response to ergonovine (<30% reduction in coronary lumen diameter) categorized as NVA patients. All 38 patients showed endothelial dysfunction manifested by a vasoconstrictive response to intracoronary acetylcholine which was mild to moderate (>10% <50% reduction in lumen diameter) in those with NVA, and severe (vasospasm occluding or nearly occluding the vessel) in those with VA. VA or NVA patients were clinically stable but had been hospitalized at least once in our institution for their anginal episodes >12 months prior to entering the study, showing lack of increase in myocardial necrosis markers in this hospitalization index. Patients with frank hypertension (=>160 mmHg), left ventricular hypertrophy, bundle branch block, valvular heart disease, previous myocardial infarction or coronary revascularization procedures were excluded. Ten consecutive patients with an uncomplicated myocardial infarction (chest pain refractory to nitroglycerin, persistent ST segment elevation and elevated enzymes) => 6 months old and without angina in the follow-up, were selected from our postmyocardial infarction outpatient clinic and constituted the CAD group.

Finally, a control group included 20 subjects from the hospital staff without apparent cardiovascular disease or major risk factors matched for age with respect to the 3 patient groups.

### Protocol

Treatment with nitrates, calcium antagonists, lipid lowering drugs or aspirin was discontinued >4 days prior to blood sampling although sublingual nitroglycerin was allowed for eventual episodes of chest pain. None of the patients, however, experienced angina during the 48 hours preceding blood sampling.

Coronary angiography was assessed by quantitative analysis (Automated coronary analysis, by Philips) by two observers unaware of the patient's condition. They evaluated the number of main epicardial coronary vessels with =>50% stenosis as well as the response to i.v. ergonovine (sequential doses 0.1 mg at 2 min intervals, up to 0.85 mg) and intracoronary acetylcholine (20, 50 and 100 micg, at 2 min intervals) in VA and NVA patients. Ejection fraction was assessed by a contrast left ventriculogram in all patients except for 4 with CAD in whom it was assessed by 2D echocardiography. The protocol was approved by the Hospital Human Ethics Committee and informed consent was obtained prior to entering the study.

### Blood sampling

Ambulatory venous blood sampling for vWF, total TFPI (t-TFPI) antigen, free TFPI (f-TFPI) antigen, PAI-1antigen and fibrinogen was performed between 08:30 and 09:30 a.m. in a fasting condition, without vascular compression and with the patient/subject lying in bed for at least the preceding 30 minutes. They were drawn >6 months after coronary angiography. The first 3 ml of blood were discarded and 9.0 ml of blood were mixed with 1.0 ml of sodium citrate (0.13 M) for vWF, t-TFPI, f-TFPI, PAI-1 and fibrinogen measurements. Samples were then centrifuged at 3,000 g for 20 minutes at 2-8°C and the citrated tube was stored at -80°C until analyzed.

### Laboratory methods

Enzyme-linked immunosorbent assays (ELISA) were used to determine plasma concentrations of vWF, t-TFPI, f-TFPI (Asserachrom, Diagnostica Stago, Asnieres, France) and PAI-1 (Tintelize plasminogen activator inhibitor - 1, Biopool, Sweden) whereas fibrinogen plasma levels were measured by the Von Clauss coagulative method. Lower limits of detection and the co-efficients of variation were 3% and 7% for vWF, 11 ng/ml and 6.0% for t-TFPI, 3.12 ng/ml and 7% for f-TFPI, 10 ng/ml and 5.3% for PAI-1, and 1.18 g/l and 6.4% for fibrinogen, respectively.

### Statistical Analysis

The Chi-square or the Fisher test were used to compare categorical variables and the normality of distribution was assessed using a normal probability plot. Analysis of variance (ANOVA) was utilized for continuous variables with normal distribution with post hoc analysis with Bonferroni correction for multiple comparisons, and the test of Kruskal-Wallis for continuous variables with abnormal distribution. The Student T test for unpaired samples was used for intergroup differences between one time measurements, and a Pearson's coefficient correlation analysis between the different parameters and the risk factors was also carried out. The possible relationship between medicines taken and levels of laboratory parameters was assessed by a univariate analysis. The analysis was performed with SPSS 13.0, data were expressed as percentage or mean mean ± SD and statistical significance was set at p < 0.05.

## Results

### Demographic and angiographic data

The 4 groups showed a similar age and gender distribution although VA patients tended to present a lower proportion of women (table 1). Incidence of arterial hypertension and smoking was comparable in the 3 patients groups, and total cholesterol and high density lipoprotein levels were also similar. Patients with VA and NVA were treated mostly with nitrates and calcium channel blockers whereas those with CAD were treated more preferentially with betablockers and aspirin (table 1).

**Table 1:** Clinical data of control subjects and patients with CAD, VA, or NVA (% and mean ± SD, ANOVA test)

	Control (20)	CAD (n:10)	VA (n:15)	NVA (n:23)	p
Age, years	54.8 ± 7.5	59.9 ± 8.9	54.5 ± 9.7	55.5 ± 9.1	0.412

Female gender, %	50	40	20	52.2	0.233

Arterial hypertension, %	0	40	53.3	56.5	0.001

Diabetes mellitus, %	0	20	0	0	0.020

Smoking, %	0	40	46.7	26	0.001

Total cholesterol, mg/dl	204 ± 31	234 ± 37	225 ± 38	223 ± 37	0.127
LDL, mg/dl	126 ± 28	158 ± 24	147 ± 23	139 ± 32	0.031
HDL, mg/dl	56 ± 12	46 ± 11	57 ± 16	56 ± 16	0.270
Triglycerides, mg/dl	108 ± 61	169 ± 95	107 ± 33	157 ± 112	0.107

Ejection fraction, %	--	55.7 ± 5.5	74.6 ± 4.4	74.1 ± 5.2	0.001

Treatment: (%)					
Nitrates	0	0	60	47.8	0.006
Beta blockers	0	70	6.7	26.1	0.003
Calcium channel blockers	0	20	86.7	56.5	0.004
ACE inhibitors	0	40.7	26.7	21.7	0.581
Aspirin	0	100	40	21.7	0.001
Statins	0	60	13.3	26.1	0.049

Culprit coronary stenosis =>70% was documented in 5/6 patients with coronary artery disease (83%).

### Coagulation factors

**Figure 1:**
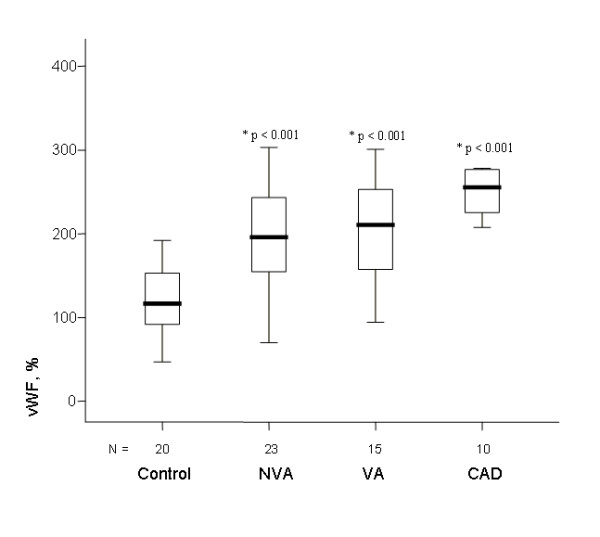
In all patient groups (NVA, VA and CAD) plasma levels of vWF levels were significantly higher than in controls (* = p value of the difference with the control group).

**Figure 2:**
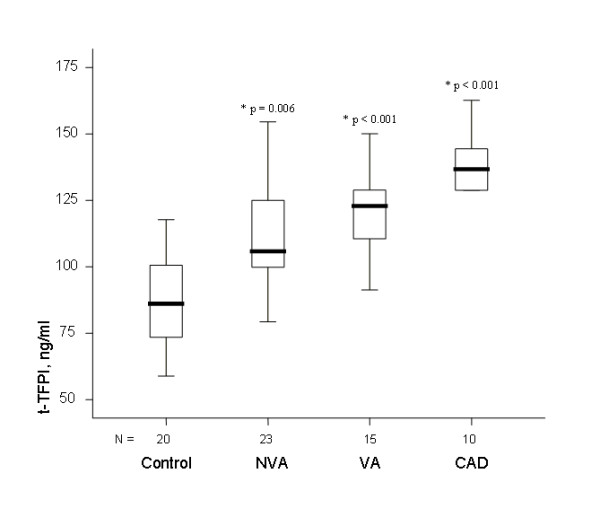
Plasma levels of t-TFPI in patients with NVA, VA, or CAD were higher than in control subjects (* = p value of the difference with the control group).

**Figure 3:**
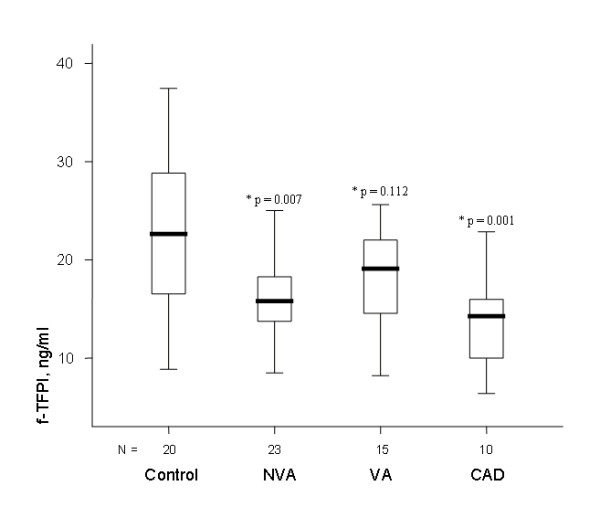
In patients with NVA or CAD, f-TFPI levels were significantly lower than in controls whereas in patients with VA there was also a similar trend (* = p value of the difference with the control group).

**Figure 4:**
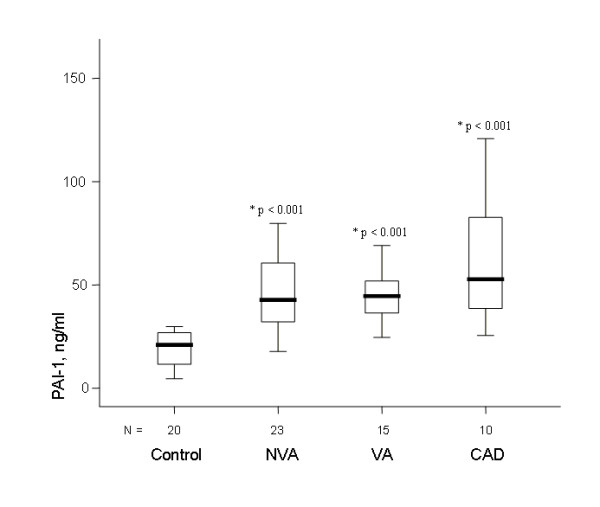
In all patient groups PAI-1levels were significantly higher than in controls (* = p value of the difference with the control group).

vWF plasma levels were higher in VA, NVA, and CAD patients than in controls

(figure 1), being higher in 93%, 96%, and 100% of patients, respectively. t-TFPI levels were also higher (figure 2) being so in 100%, 87% and 100%, respectively). In contrast, f-TFPI levels were lower in the 3 groups (figure 3), being lower in 67%, 87%, and 90% of patients, respectively, whereas fibrinogen plasma levels tended to be higher in the 3 groups than in the control group but differences were not statistically significant. Moreover, PAI-1 plasma levels were significantly higher in VA, NVA and CAD groups than in controls (figure 4), being higher in 100%, 91% and 100% of patients, respectively, and were significantly although modestly correlated with t-TFPI and vWF plasma levels (r:0.329, p = 0.008, and r:0.299, p = 0.015

Age was correlated with vWF (r:0.365, p = 0.002) and cholesterol levels with t-TFPI (r:0.449, p = 0.001) and PAI-1 (r:0.361, p = 0.004). Treatment with nitrates, ACE inhibitors, beta blockers calcium channel blockers or aspirin did not seem to influence plasma levels of hemosthatic parameters since in a univariate analysis patients treated with these agents showed comparable levels than those untreated. Patients treated with statins, however, had higher levels of PAI-1 (68.5 ± 37.4 vs 44.8 ± 25 ng/ml, p = 0.030) and lower levels of t-TFPI (114.6 ± 23.8 vs 130.8 ± 20.6 ng/ml, p = 0.031) than untreated patients likely because of the alluded correlation between these parameters and cholesterol levels.

### Follow-up

During 9 years (108 ± 10 months) there were no cardiac deaths or myocardial infarction in any of the 38 patients with VA or NVA. Six VA patients (40%) and 17 with NVA (74%), however, continued to present angina although with less frequency and intensity than before entering the study.

## Discussion

Our study documented that patients with stable VA or NVA had higher levels of vWF, t-TFPI and PAI-1 than a control group and lower levels of f-TFPI. Moreover, their values were comparable to patients with asymptomatic CAD. However, although the association of this prothrombotic profile with coronary endothelial dysfunction might have facilitated development of coronary thrombosis, none of our patients presented a myocardial infarction during an 9 years follow-up which likely suggest the concurrence of protective mechanisms.

### vWF

Plasma levels of vWF have not been previously investigated in VA patients and 3 studies have analyzed vWF levels in patients with NVA (26-28) with variable results. Thus, Botker [26] and Kolasinska [27] found vWF levels in NVA patients similar to a control group but they represented a different cohort than ours for they had only effort angina while ours presented rest and, often, mixed angina. In contrast, Lin et al. reported increased vWF levels in NVA patients [28]. These investigators [27,28] included a much greater proportion of male patients, 64% [27] and >70% [28], than in our study and than in previous larger NVA series [20-22] also suggesting a different patient population. In addition, they did not assess - angiographically - the coronary endothelial function of their patients [26-28].

vWF is a glycoprotein that is critical for platelet adhesion and aggregation to exposed subendothelium, and plays a major role in thrombus formation [29]. In fact, increased vWF levels predict adverse cardiac events and recurrence of myocardial infarction in patients with acute coronary syndromes [14,17].

### Total and free TFPI

There are no previous studies measuring t-TFPI in patients with VA or NVA. Thus, our observation of increased plasma t-TFPI levels in these patients with respect to a control group constitute a novel finding. This is of interest since increased TFPI levels have been reported in patients with unstable angina or acute myocardial infarction [10,11] suggesting a possible causal role in coronary thrombosis. Indeed, TFPI is the most important inhibitor of tissue factor which is the principal determinant of thrombus formation after plaque rupture [30], and it is thought that increased levels represent an attempt to balance the procoagulant effects of increased tissue factor levels [9,31].

Of additional interest, our patients with VA or NVA showed lower levels of f-TFPI than control subjects. There is only one study where f-TFPI plasma levels were investigated in patients with atypical chest pain and non significant coronary stenosis, and were found to be similar to patients with stable angina [10]. In this study, however, patients were no diagnosed of angina and hence could be different than ours, who presented with typical spontaneous (rest) angina. Furthermore, there was no control group and coronary endothelial function was not assessed [10]. The significance of abnormal plasma levels of f-TFPI, however, remains controversial. Thus, while Soejima [10] reported that increased levels of f-TFPI in patients with unstable angina were associated with increased risk of unfavorable outcomes, Morange demonstrated that reduced rather than increased f-TFPI plasma level was an independent risk factor for myocardial infarction in 10.000 healthy men being an even better predictor when associated with increased levels of vWF [32]. Likewise, reduced f-TFPI levels have been recently linked to intravascular thrombosis in patients with a stroke [33] or deep vein thombosis [34]. However, the reduced levels in our VA and NVA were not associated with development of coronary thrombosis.

### PAI-1

VA patients presented elevated PAI-1 levels and similar observations were made by Masuda [35] and Yamaguchi [36]. In contrast, Misumi et al. showed increased PAI-1 levels in VA patients but only following coronary spasm and not at baseline [37]. Increased PAI-1 levels were also documented in our NVA patients and, in the only existing report [27], NVA patients tended also to show higher plasma levels than a control group, particularly during exercise, although the differences reached no statistical significance [27]. This study, however, failed to provide angiographic evidence of endothelial dysfunction and had an unusually high proportion of males, 64% [27].

PAI-1 is released by activated platelets and endothelial cells and effectively inhibits fibrinolysis [38] by neutralizing tissue plasminogen activator. Increased plasma levels have been found in patients with unstable angina or acute myocardial infarction [15,35] and seem to precede occurrence or recurrence of myocardial infarction [12-14].

### Endothelial dysfunction and myocardial infarction

Despite reduced f-TFPI and increased vWF and PAI-1 levels - all apparent procoagulant factors and markers of endothelial dysfunction - myocardial infarction did not occur in our series and has not been a frequent finding in the follow-up of larger series of patients with VA without significant stenosis [18,19] or patients with NVA [20-22]. In some contemporary series, however, adverse outcomes have been more frequently reported in NVA patients with proven endothelial dysfunction [23-25].

Development of a thrombotic coronary occlusion in CAD patients appears to be independent of the severity of coronary stenosis since it often occurs in non significantly stenosed vessels [8,39] and may even develop over an endothelial erosion [7]. Thus, since patients with VA or NVA often share conventional risk factors and some degree of atherosclerosis with those with CAD, it is conceivable that further derangements in their procoagulant profile could supervene during an unstable phase - intense emotional stress [40] - thereby enhancing the risk of coronary thrombosis. In this respect, Buggiardini has recently reported a low incidence of myocardial infarction during a 10 year follow-up in 42 patients with NVA, 5%, but that was higher in those with a vasoconstrictive response to acetylcholine [25]. By and large, however, the remarkably low incidence of thrombotic events despite the correlation between cholesterol levels and t-TFPI and PAI-1 shown in our study points to protective mechanisms - possibly genetically mediated - against development of complicated artheriosclerotic plaques. Indeed, in view of the reduced use of statins in VA and NVA patients it is unlikely that cholesterol levels and their positive interaction with prothromboptic factors would decline in the follow-up. Similarly, the infrequent use of platelet antiaggregants would further lower the possibility of a pharmacological protection.

### Limitations

Intracoronary ultrasonographic studies to clarify the proportion of NVA patients with a completely normal vascular walls, intimal thickening, or small atherosclerotic plaques [41], were not available in our study. Nevertheless, all these patients showed a <50% culprit stenosis, were well characterized clinically, and had a proper assessment of their coronary endothelial dysfunction. We admit also that the reduced sample size as well as the lack of measurements of prothrombotic markers in the follow-up limits the clinical implications with respect to the risk of coronary thrombotic events although follow-up was long and larger series had shown similar results [18-21]. In addition, even though we attempted to avoid blood sampling during inflammatory processes given their potential interaction with coagulation parameters, we can not totally rule out such an effect.

## Conclusions

Our findings indicate that stable patients with VA or NVA and angiographically documented coronary endothelial dysfunction present increased levels of vWF, t-TFPI and PAI-1, and reduced levels of f-TFPI. They contribute to the understanding of these poorly defined syndromes and suggest that this association of coronary endothelial dysfunction with a prothrombotic state might eventually facilitate coronary thrombosis, particularly under stressful conditions [40]. However, the lack of development of myocardial infarction - also seen in larger series [18-22] - would confirm that this is an unusual complication. Moreover, since less than 1/3 of our patients were treated with statins and platelet antiaggregants, it is tempting to speculate that they were *naturally *(genetically) protected from coronary plaque rupture and subsequent thrombosis.

## List of abbreviations

VA: vasospastic angina; NVA: non vasospastic angina; CAD: coronary artery disease; vWF: von Willebrand factor; t-TFPI: total tissue factor pathway inhibitor; f-TFPI: free tissue factor pathway inhibitor; PAI-1: plasminogen activator inhibitor-1.

## Competing interests

The authors declare that they have no competing interests.

## Authors' contributions

JF: has contributed to conception and design, acquisition of data, analysis and interpretation of data; has also been involved in drafting the manuscript and has given final approval of the version to be published.

JM: has made substantial contributions to conception and design, and has participated in the analysis and interpretation of data and revising it critically for important intellectual content. She has also given final approval of the version to be published.

ED: has made substantial contributions to conception, design, acquisition of data, analysis and interpretation of data, and has been involved in revising the manuscript critically for important intellectual content. He has also given final approval of the version to be published.

BM: has made substantial contributions to acquisition of data, and has been involved in drafting the manuscript and revising it critically for important intellectual content. She has also given final approval of the version to be published.

EN: has made substantial contributions to acquisition, analysis and interpretation of data. She has also been involved in revising the manuscript critically for important intellectual content and has given final approval of the version to be published.

JC: has made substantial contributions to acquisition, analysis and interpretation of data. She has also been involved in revising the manuscript critically for important intellectual content and has given final approval of the version to be published.

DGD: has made substantial contributions to alysis and interpretation of data. He has also been involved in revising the manuscript critically for important intellectual content and has given final approval of the version to be published.
